# Technology-Supported Guidance Models Stimulating the Development of Critical Thinking in Clinical Practice: Protocol for a Mixed Methods Systematic Review

**DOI:** 10.2196/25126

**Published:** 2021-01-19

**Authors:** Jaroslav Zlamal, Edith Roth Gjevjon, Mariann Fossum, Marianne Trygg Solberg, Simen Alexander Steindal, Camilla Strandell-Laine, Marie Hamilton Larsen, Fredrik Solvang Pettersen, Andréa Aparecida Gonçalves Nes

**Affiliations:** 1 Lovisenberg Diaconal University College Oslo Norway; 2 University of Agder Kristiansand Norway; 3 Novia University of Applied Sciences Åbo Finland

**Keywords:** critical thinking, technology, guidance models, nursing education, clinical practice

## Abstract

**Background:**

Critical thinking is an essential skill that nursing students need to develop. Technological tools have opened new avenues for technology-supported guidance models, but the challenges and facilitators of such guidance models, as well as how they stimulate the development of critical thinking, remain unclear.

**Objective:**

We developed a protocol for a mixed methods systematic review to investigate the use of technology-supported guidance models that stimulate the development of critical thinking in nursing education clinical practice.

**Methods:**

A convergent integrated design following the Joanna Briggs Institute Manual for Evidence Synthesis will be employed. A pair of authors will select the articles by screening titles and abstracts, and the methodological quality of the articles included in the review will be assessed by a pair of authors according to checklists for specific study designs. The data will be extracted using the standardized Joanna Briggs Institute mixed methods data extraction form and following a convergent integrated approach. The thematic synthesis for data transformation will be used.

**Results:**

Development of a comprehensive systematic search strategy was completed in October 2020. The database searches were performed on October 21, 2020. As of January 2021, analysis and synthesis is ongoing. Completion of this review is expected by January 2021.

**Conclusions:**

By combining evidence from studies with varied methodological approaches, the results should provide broad insight into the use of technology-supported guidance models for clinical practice in nursing education with a focus on the development of nursing students’ critical thinking. The results of this mixed methods systematic review can also be used to develop or improve current technology-supported guidance models for clinical practice in nursing education.

**International Registered Report Identifier (IRRID):**

PRR1-10.2196/25126

## Introduction

### Background

Critical thinking is an essential element of the skill set of all health professionals as it enables them to address complex problems and make informed evidence-based decisions [1,2].

This is especially true in the nursing profession as nurses provide complex, prolonged care to a diverse group of patients [3]. Thus, critical thinking is a prerequisite for being able and enabled to provide safe qualified care [4,5], and it is a key component of undergraduate nursing students’ emerging competencies [6,7]. Clinical practice is an essential part of the nursing curriculum [8] and is the setting best suited to developing nursing students’ necessary skills, such as critical thinking, for their future role as nurses [7].

Critical thinking is a broad concept, and terms such as clinical decision making, analytical thinking, creative thinking, problem solving, reflective thinking, diagnostic reasoning, and clinical judgement are often used interchangeably to describe critical thinking [9]. Nursing has often adopted definitions of critical thinking that are different from those used in other disciplines [10]. In the consensus definition of critical thinking, cognitive skills such as information seeking, knowledge transformation, logical reasoning, and application of standards are highlighted [11].

In addition to cognitive skills, self-awareness, creativity, and risk taking are also deemed important [10]. According to Facione [12], critical thinking is a judgement that is purposeful and self-regulatory and that results in a process of interpretation, analysis, evaluation, and inference.

Clinical practice is an ideal context in which to develop critical thinking [5,13]. This skill is facilitated by a nurse preceptor (a registered nurse working in clinical practice) who, by posing questions, examining problems, and contemplating different ways of thinking about a patient’s situation, stimulates the development of students’ critical thinking [14,15]. Other strategies to stimulate critical thinking among nursing students involve the use of problem-based learning, case-based learning, and concept mapping [9]. A guidance model (a framework of procedures, meetings, and cooperation) between health care and educational institutions is also often used to facilitate the acquisition of nursing students’ competencies in clinical practice [16].

Novel technologies afford new opportunities for supporting nursing students in clinical practice and developing their critical thinking [17,18], but the use of educational technologies in nursing education lacks a solid evidence base [19], and a wide range of technological tools has been adopted without clear recommendations about their use in nursing education [20].

In a meta-analysis, Ismail et al [17] reviewed available research on how technological tools, such as mobile technology, might improve nursing students’ critical thinking. Most of the studies in that meta-analysis [17] reported that the use of mobile technology improved critical thinking but that the actual effectiveness of mobile technology in the development of critical thinking remains unclear. Mobile apps incorporate several strategies, such as cooperative learning and problem-based learning, but the mobile apps and strategies in the meta-analysis were not situated in the clinical education and guidance of nursing students.

Another study, conducted by Lee et al [19], reviewed the use of mobile technology in nursing education and noted that mobile technology is still immature in this field; technology is often used for quickly accessing evidence-based information, submitting various requirements to educational institutions, and communicating with nurse educators, yet its potential to support the development of competencies is unclear.

Regarding guidance models, Jayasekara et al [21] identified 4 clinical educational models used in clinical practice: the clinical education unit model, the standard facilitation model, the collaborative clinical placement model, and the mentor-arranged clinical placement model. None of these approaches includes the use of technological tools.

In conclusion, the existing systematic reviews and meta-analyses are limited to the development of critical thinking in nursing education (both in and outside clinical practice) without the use of technological tools [9,22,23]. Consequently, a systematic literature review is needed that focuses on critical thinking as a competency and on its development in clinical practice in nursing education through guidance and the use of technology. This focus can be informed by evidence from various types of studies; therefore, a mixed methods systematic review is appropriate. Mixed methods systematic reviews are suitable for answering complex questions because the methodology allows the inclusion, integration, and discussion of qualitative, quantitative, or mixed methods primary studies [24]. To the best of our knowledge, no earlier reviews or protocols of reviews have appraised existing studies on technology-supported guidance models that aim to stimulate critical thinking among nursing students in clinical practice.

### Aim

This study outlines a mixed methods systematic review with an overall aim of synthesizing available knowledge about various technology-supported guidance models that employ technological tools in clinical practice to stimulate the development of critical thinking among nursing students.

### Review Questions

Which technology-supported guidance models are used to stimulate the development of critical thinking in the context of clinical practice in nursing education?

What is known about the challenges and facilitators of such technology-supported guidance models?

## Methods

### Design

This mixed methods systematic review will be guided by the Joanna Briggs Institute Manual for Evidence Synthesis and will have a convergent integrated design [24]. The convergent integrated design involves the transformation, integration, and synthesis of data from primary qualitative, quantitative, or mixed methods studies [24]. The reporting of this systematic mixed methods review protocol is guided by the PRISMA-P (Preferred Reporting Items for Systematic Reviews and Meta-Analyses) checklist [25].

### Eligibility Criteria

We will include evidence that addresses preregistration or undergraduate nursing students in clinical practice; further details on the inclusion and exclusion criteria are provided in Table 1.

**Table 1:** Inclusion and exclusion criteria.

Criterion	Inclusion	Exclusion
Study population	Preregistration nursing students or undergraduate nursing students	Nursing students studying at the master’s or PhD degree level; postregistration nursing students; student paramedics; students of midwifery, physiotherapy or occupational therapy; medical students; dental students
Phenomenon of interest	Technological tools used in clinical practice and technology-assisted guidance models or technology-supported guidance models or guidance models or mentoring or tutoring or preceptorship in clinical practice or clinical educational models	Technology-assisted guidance models; clinical educational models; guidance models; mentoring, tutoring, or preceptorship outside clinical practice, in clinical labs and as a preparation for clinical practice; simulation or technology use in conjunction with simulation.
Context	Clinical practice in hospitals, nursing homes, community health care, or other health care institutions and settings	Outside clinical practice, such as in classes for preparation for clinical practice, simulation sessions and training in a clinical laboratory
Type of study	Qualitative, quantitative, and mixed methods studies using experimental, quasi-experimental, or nonexperimental design published in peer-reviewed journals	Any type of systematic or nonsystematic review, non–peer-reviewed articles, conference proceedings, comments or opinion articles, official guidelines, national nursing curriculums, editorials, abstracts and doctoral theses
Type of outcome	Critical thinking, clinical decision making, analytical thinking, creative thinking, problem solving, reflective thinking, diagnostic reasoning, clinical judgement	All other outcomes

### Search Strategy

A systematic, comprehensive search strategy will be built through an initial search in MEDLINE and CINAHL by an experienced research librarian, the first author, and the last author using subject terms, Medical Subject Heading terms, CINAHL headings, and text words. The search strategy includes terms chosen based on an initial search and discussion within the review team. The search will be limited to publications in English, Slovak, Hungarian, Czech, Spanish, Portuguese, Finnish, Norwegian, Swedish, and Danish. To capture the studies most relevant to current and emerging technologies in nursing education, the search strategy will be limited to articles published from January 1, 2010 through December 31, 2020.

The search strategy will be tested and retested [26] in the initial databases before it is peer reviewed by a second research librarian. The search strategy will then be applied to CINAHL, Cochrane Trials, Embase, ERIC, MEDLINE, PsycINFO, and Web of Science. An example of the MEDLINE search strategy is provided in Multimedia Appendix 1.

In addition, forward and backward reference searches will be conducted. A search for unpublished studies and other grey literature will not be included. The rationale for not conducting a search for grey literature is the lack of a standard, accepted systematic procedure for such searches [27]. This lack of a standard procedure, combined with the surfeit of sources of grey literature, could produce a search with unsystematic and random results.

### Data Management

Records will be managed through EndNote (Clarivate Analytics) [28], and Rayyan (Qatar Computing Research Institute) [29] will be used to facilitate the screening, blinding, organization, and storage of the publications for the study selection process.

### Selection Process

**Figure 1:**
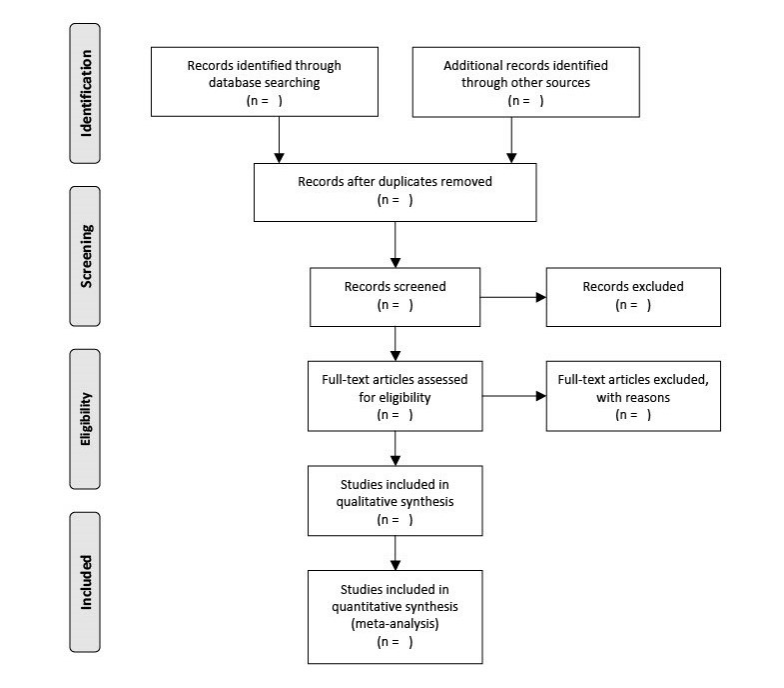
PRISMA [29] flow diagram of the selection process that will be used.

Titles and abstracts will be screened independently by pairs of authors (AAGN and JZ, ERG and MF, MHL and CS-L, SAS and MTS) based on the inclusion and exclusion criteria for the review. From this selection, the full-text articles will be assessed independently by pairs of authors against the inclusion and exclusion criteria for the review. The final decision on whether to include or exclude articles will be made by consensus between the team of authors. An overview of the selection process that will be used is shown in a PRISMA [30] flow diagram (Figure 1).

### Assessment of Methodological Quality

Studies eligible for inclusion will be critically assessed for their methodological quality. The critical assessment will be conducted by pairs of authors (AAGN and JZ, ERG and MF, MHL and CS-L, SAS and MTS) according to checklists specific to the study design. The tools used to assess the methodological quality of the studies are shown in Table 2. If required, authors will be contacted for additional data or to provide missing data. During the review process, if a pair of authors disagrees on the assessment of the methodological quality of the articles, either the disagreement will be resolved by discussion between the pair, or another author (the first or last author) will independently appraise the quality of the study.

All studies, regardless of the results of the assessment of methodological quality, will be included in the data extraction and synthesis, but the results of the assessment of methodological quality will be elaborated on in discussion, and the results will be displayed in appropriate tables.

**Table 2:** Checklist for the assessment of methodological quality.

Type of study	Checklist or tool
Cohort studies	Joanna Briggs Institute Checklist for Cohort Studies [31]
Case-control studies	Joanna Briggs Institute Checklist for Case Control Studies [32]
Qualitative studies	Joanna Briggs Institute Checklist for Qualitative Research [33]
Cross-sectional studies	Appraisal Tool for Cross-Sectional Studies [34]
Mixed methods studies	Mixed Methods Appraisal Tool [35]
Quasi-experimental studies	Joanna Briggs Institute Checklist for Quasi-Experimental Studies [36]
Randomized controlled trials	Cochrane Risk-of-Bias Tool [37]

### Data Extraction and Data Items

Quantitative and qualitative data will be extracted by pairs of authors (AAGN and JZ, ERG and MF, MHL and CS-L, SAS and MTS) from studies that meet the inclusion criteria using the standardized Joanna Briggs Institute mixed methods data extraction form and following a convergent integrated approach [24]. The extracted data will include population, phenomenon of interest, type of study, methods, context, time period and outcomes. Quantitative data will include percentage or average (for descriptive studies) and significant and nonsignificant results (for analytical studies). Qualitative data will include themes and subthemes with, for example, supporting quotations from participants. Qualitative data will be assigned a level of credibility (unequivocal, credible, or not supported) according to the Joanna Briggs Institute Manual for Evidence Synthesis [24].

### Outcomes

The primary outcome is critical thinking, as defined by Facione [12], as well as synonyms of the term *critical thinking*, as defined in Table 1.

### Data Transformation, Synthesis, and Integration

To facilitate combining qualitative and quantitative data, quantitative data will first be transformed into qualitized data. The process of qualitizing data refers to converting quantitative data into themes through textual description of quantitative data in relation to the review question [24]. This will be accomplished by thematic analysis [24]. NVivo (version 12; QSR International) [38] will be used to store and synthesize data. We will use thematic synthesis for data synthesis and integration. Qualitized and qualitative data are assembled according to similar meanings [24]; coding themes are coded and codes are grouped by similarity to develop encompassing themes that will answer the review question. In that manner,

### Confidence in the Cumulative Evidence

According to the Joanna Briggs Institute Manual for Evidence Synthesis, the assessment of the certainty of evidence using GRADE (Grading of Recommendations Assessment, Development and Evaluation) is not recommended for mixed methods systematic reviews [24].

## Results

A comprehensive systematic search strategy was developed by a research librarian in MEDLINE and CINAHL and reviewed by a second research librarian (completed in October 2020). Initial database searches were performed on October 21, 2020, and resulted in 7307 publications. After the removing 3861 duplicates, we began screening the titles and abstracts of 3446 publications in addition to conducting manual searches and contacting researchers in this field. From the results of this selection, we will assess the full-text articles. We anticipate that the review will be completed by January 2021. Table 3 provides a detailed timeline of the stages of this mixed methods review. The results should clarify the feasibility and reliability of the technological guidance models used in clinical nursing education.

**Table 3:** Timeline of completion of the stages of mixed methods review.

Stage of the review	Date of completion
Building a comprehensive search strategy	October 2020
Application of the search strategy in databases	November 2020
Screening search results from databases	November 2020
Assessment of methodological quality	December 2020
Data extraction	December 2020
Data transformation, synthesis, and integration	January 2021

## Discussion

### General

In this mixed methods systematic review, we will discuss the contribution of technology to guidance models that are employed in nursing education clinical practice settings, focusing on the stimulation and development of critical thinking among nursing students.

### Significance of the Results

Technology is an important part of nursing education that has the potential to significantly improve it, especially in clinical practice [18]. Earlier research shows that various technological tools have been implemented in nursing education with varied degrees of use, but their implementation and use in nursing clinical practice sometimes appear unsystematic [20]. This study’s results will enable the improvement of current or the further development of new technology-supported guidance models, which may benefit nursing students, nurse educators, and health care institutions.

### Limitations of the Review

One of the limitations of this study is the exclusion of unpublished studies and other grey literature. Such material can potentially benefit a systematic review, but the challenges of searching for grey literature and including its findings [27] outweigh its benefits for this study. By choosing a mixed methods systematic review with an integrated convergent design, however, we have facilitated a comprehensive synthesis of peer-reviewed empirical evidence. This approach makes possible broad novel insight into the use, challenges, and facilitators of technology-supported guidance models in nursing clinical practice [24].

## Appendix

Multimedia Appendix 1 Example of the MEDLINE search strategy.
