# Supplementary material for: Technology-Supported Guidance Models Stimulating the Development of Critical Thinking in Clinical Practice: Protocol for a Mixed Methods Systematic Review
Source: JMIR Res Protoc. 2021 Jan 19;10(1):e25126. doi: 10.2196/25126 (PMC7854029; doi:10.2196/25126)
Supplement: Multimedia Appendix 1 [file resprot_v10i1e25126_app1.docx]

Multimedia Appendix 1. Example of the MEDLINE search strategy.

Database(s): Ovid MEDLINE(R) and Epub Ahead of Print, In-Process & Other Non-Indexed Citations and Daily 1946 to October 12, 2020

Search Strategy:

| # | Searches | Results |
| --- | --- | --- |
| 1 | ((nurse or nursing or nurses) adj5 (student* or education or undergrad* or under-grad* or bachelor* or baccalaur* or preregistration or preregistration)).tw,kf. | 54371 |
| 2 | education, nursing/ | 32842 |
| 3 | education, nursing, baccalaureate/ | 18254 |
| 4 | Students, Nursing/ | 24711 |
| 5 | Nursing Education Research/ | 8902 |
| 6 | 1 or 2 or 3 or 4 or 5 | 89716 |
| 7 | (student* adj3 (practic* or practis* or placement* supervis* or clerkship* or apprenticeship*)).tw,kf. | 8614 |
| 8 | (clinic* adj3 (practic* or practis* or placement* or supervis* clerkship* or apprenticeship* or setting* or pedagog* or teach* or learn* or educat* or training*)).tw,kf. | 357247 |
| 9 | coach*.tw,kf. | 16501 |
| 10 | mentor*.tw,kf. | 17740 |
| 11 | preceptor*.tw,kf. | 4387 |
| 12 | clinical clerkship/ | 5142 |
| 13 | mentoring/ | 1869 |
| 14 | Mentors/ | 10901 |
| 15 | Preceptorship/ | 5140 |
| 16 | 7 or 8 or 9 or 10 or 11 or 12 or 13 or 14 | 404474 |
| 17 | Telecommunications/ | 4837 |
| 18 | Telephone/ | 11885 |
| 19 | Cell phone/ | 8637 |
| 20 | Videoconferencing/ | 1626 |
| 21 | Webcasts as topic/ | 348 |
| 22 | Wireless technology/ | 3627 |
| 23 | Text messaging/ | 3029 |
| 24 | Technology/ | 9672 |
| 25 | Educational technology/ | 1529 |
| 26 | Mobile Applications/ | 6371 |
| 27 | Computers, handheld/ | 3645 |
| 28 | Smartphone/ | 4750 |
| 29 | Internet/ | 73607 |
| 30 | (mobile* or phone* or phoning or telephon* or cellphon* or smartphon* or iphon* or android*).tw,kf. | 209604 |
| 31 | electronic*.tw,kf. | 298097 |
| 32 | digital*.tw,kf. | 158169 |
| 33 | video*.tw,kf. | 138815 |
| 34 | web*.tw,kf. | 176909 |
| 35 | internet.tw,kf. | 59156 |
| 36 | online.tw,kf. | 133179 |
| 37 | app*1.tw,kf. | 39987 |
| 38 | app-base*.tw,kf. | 717 |
| 39 | telecommunicat*.tw,kf. | 4739 |
| 40 | tele-communicat*.tw,kf. | 14 |
| 41 | tablet*.tw,kf. | 57355 |
| 42 | ((portable or handheld or hand-held) adj1 computer*).tw,kf. | 1008 |
| 43 | etutor*.tw,kf. | 2 |
| 44 | e-tutor*.tw,kf. | 10 |
| 45 | ((short or text or instant) adj1 messag*).tw,kf. | 6067 |
| 46 | texting.tw,kf. | 1038 |
| 47 | sms.tw,kf. | 6690 |
| 48 | eportfolio*.tw,kf. | 61 |
| 49 | e-portfolio*.tw,kf. | 141 |
| 50 | (wireless adj2 communicat*).tw,kf. | 2003 |
| 51 | pda.tw,kf. | 13037 |
| 52 | personal digital assistant*.tw,kf. | 1041 |
| 53 | "virtual session*".tw,kf. | 17 |
| 54 | 17 or 18 or 19 or 20 or 21 or 22 or 23 or 24 or 25 or 26 or 27 or 28 or 29 or 30 or 31 or 32 or 33 or 34 or 35 or 36 or 37 or 38 or 39 or 40 or 41 or 42 or 43 or 44 or 45 or 46 or 47 or 48 or 49 or 50 or 51 or 52 or 53 | 1193131 |
| 55 | 6 and 16 and 54 | 1763 |
| 56 | limit 55 to yr="2010 -Current" | 1368 |
| 57 | limit 56 to (comment or editorial or letter) | 6 |
| 58 | 56 not 57 | 1362 |
